# Cytotoxicity and genotoxicity of bacterial magnetosomes against human retinal pigment epithelium cells

**DOI:** 10.1038/srep26961

**Published:** 2016-06-01

**Authors:** Lei Qi, Xiujuan Lv, Tongwei Zhang, Peina Jia, Ruiying Yan, Shuli Li, Ruitao Zou, Yuhua Xue, Liming Dai

**Affiliations:** 1 Institute of Advanced Materials for Nano-Bio Applications, School of Ophthalmology & Optometry, Eye Hospital, Wenzhou Medical University, 270 Xueyuan Xi Road, Wenzhou, Zhejiang 325027, China; 2 Biogeomagnetism Group, Paleomagnetism and Geochronology Laboratory, Key Laboratory of Earth and Planetary Physics, Institute of Geology and Geophysics, Chinese Academy of Sciences, Beijing 100029, China; 3 France-China Bio-Mineralization and Nanostructures Laboratory, Chinese Academy of Sciences, Beijing 100029, China; 4 Department of Microbiology, College of Biological Sciences, China Agricultural University, Beijing, 100193, China; 5 Center of Advanced Science and Engineering for Carbon (Case4Carbon), Department of Macromolecular Science and Engineering, Case Western Reserve University, Cleveland, OH 44106, United States

## Abstract

A variety of nanomaterials have been developed for ocular diseases. The ability of these nanomaterials to pass through the blood-ocular barrier and their biocompatibility are essential characteristics that must be considered. Bacterial magnetosomes (BMs) are a type of biogenic magnetic nanomaterials synthesized by magnetotactic bacteria. Due to their unique biomolecular membrane shell and narrow size distribution of approximately 30 nm, BMs can pass through the blood-brain barrier. The similarity of the blood-ocular barrier to the blood-brain barrier suggests that BMs have great potential as treatments for ocular diseases. In this work, BMs were isolated from magnetotactic bacteria and evaluated in various cytotoxicity and genotoxicity studies in human retinal pigment epithelium (ARPE-19) cells. The BMs entered ARPE-19 cells by endocytosis after a 6-h incubation and displayed much lower cytotoxicity than chemically synthesized magnetic nanoparticles (MNPs). MNPs exhibited significantly higher genotoxicity than BMs and promoted the expression of Bax (the programmed cell death acceleration protein) and the induction of greater cell necrosis. In BM-treated cells, apoptosis tended to be suppressed via increased expression of the Bcl-2 protein. In conclusion, BMs display excellent biocompatibility and potential for use in the treatment of ocular diseases.

Magnetic nanoparticles are widely used in biomedical applications^1^,^2^,^3^. However, the biocompatibility of chemically synthesized magnetic nanoparticles (MNPs) usually must be improved by complicated chemical modifications. Bacterial magnetosomes (BMs), a type of biosynthesized magnetic nanoparticle, have attracted great interest due to their unique nanostructure, encapsulation by a biomolecular membrane, and narrow size distribution^4^. BMs have been investigated for use in medical and diagnostic applications, such as magnetic resonance imaging (MRI), drug delivery, and hyperthermia^5^,^6^,^7^. In contrast to chemically synthesized MNPs, BMs are synthesized by magnetotactic bacteria and are generally composed of Fe_3_O_4_ or Fe_3_S_4_, with a size range of 30 to 120 nm^8^. Large-scale culture of *Magnetospirillum gryphiswaldense* MSR-1 has made high-yield and low-cost production of Fe_3_O_4_-containing BMs feasible^9^.

The eye is a fragile organ and is protected by the blood-ocular barrier, which is created by the endothelium of the capillaries of the retina and iris, ciliary epithelium and retinal pigment epithelium. However, the blood-ocular barrier also prevents most drugs from entering the eye and hinders targeted medical therapies (e.g., ocular tumor-related treatments)^10^. Han *et al.*^11^ reported that BMs can pass through the blood-brain barrier, which is similar in structure and function to the blood-ocular barrier, and carry genes to treat brain tumors. Intravitreal injection of chemically synthesized MNPs in mice^12^, *Xenopus* and zebrafish^12^,^13^ does not damage retinal structure. Therefore, BM-based nanomaterials have great potential for use as new therapeutic approaches in ophthalmology.

The biocompatibility of BMs must be assessed prior to use in ophthalmology applications, but studies of the biocompatibility of BMs have been limited. In a preliminary study, Sun *et al.*^14^ demonstrated that BMs induced little pathological damage in important organs in rats and little cytotoxicity against H22, HL60 and EMT-6 cells. Liu *et al.*^15^ reported that BMs mainly accumulated in the rat spleen and liver and were completely excreted in 42 days. The ocular biocompatibility of BMs has not been reported. Human ocular exposure to BMs is unavoidable, particularly among patients using BM-based medicines and workers handling these materials and the wastes of BM products, emphasizing the importance of evaluating the ocular biocompatibility of BMs. Here, we perform a detailed study of the biocompatibility of BMs in terms of both intraocular cytotoxicity and genotoxicity. For comparison, commercial MNPs with similar morphologies, sizes and surface groups were also evaluated as a control.

## Results and Discussion

### Characterization of BMs and MNPs

BMs were isolated from MSR-1. The UV absorption of the extracted supernatants at 260 and 280 nm decreased at each step until they reached zero (Fig. S1a), and no proteins were detected at the fifth step by sodium dodecyl sulfate-polyacrylamide gel electrophoresis (SDS-PAGE) (Fig. S1b), indicating that the BMs were free from other cellular protein fractions. However, “hard” membrane proteins were abundant in the BM membranes, indicating the presence of magnetosome membranes (Fig. S1). By contrast, protein was not detected on the surface of MNPs by SDS-PAGE (Fig. S1c).

**Figure 1:**
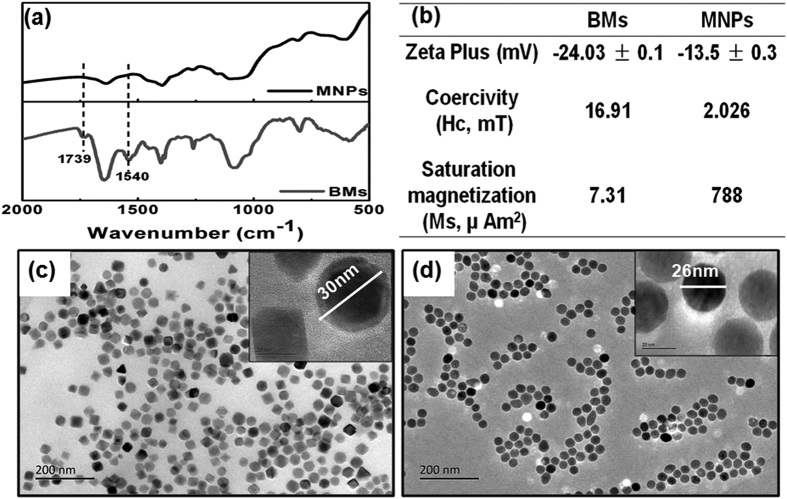
Characterization of the BMs and MNPs. (**a**) FTIR spectra of BMs and MNPs. (**b**) Zeta Plus and magnetic hysteresis values of the BMs and MNPs. (**c**,**d**) HRTEM images of the BMs (**c**) and MNPs (**d**).

The surfaces and cores of the BMs and MNPs were further characterized by Fourier transform infrared spectroscopy (FTIR), zeta potential, magnetic hysteresis curves and high-resolution transmission electron microscopy (HRTEM). The MNPs were coated by organic layers containing amino groups (SIGMA-ALDRICH, CAS 1317-61-9). The FTIR spectra (Fig. 1a) indicated that the BMs and MNPs were both Fe-O-based materials (585 cm^−1^) embedded with amino groups (1020–1360 cm^−1^ and 1550–1660 cm^−1^). However, the BMs had two additional peaks at 1739 and 1540 cm^−1^, corresponding to carbonyl and amide groups, respectively. The zeta potentials of the BMs and MNPs were −24.03 ± 0.1 and −13.5 ± 0.3, respectively (Fig. 1b). The magnetic hysteresis curves of the BMs and MNPs were obviously different (Fig. S2, Fig. 1b). The curves indicated that the BMs were typical, single-domain magnetite, with a coercivity (Hc) value of 16.91 mT (greater than 0) and a saturation magnetization (Ms) value of 7.31 μAm^2^. However, the Hc value of the MNPs was close to 0, indicating that the MNPs were intermediate between single-domain magnetite and super paramagnetic. We further observed the BMs and MNPs by HRTEM. The BMs were subspheroidal, and the MNPs were spherical; both displayed good dispersity and similar particle diameters (approximately 30 nm) (Fig. 1c,d).

### Cytotoxicity of BMs and MNPs

#### Cell morphology (TEM)

**Figure 2:**
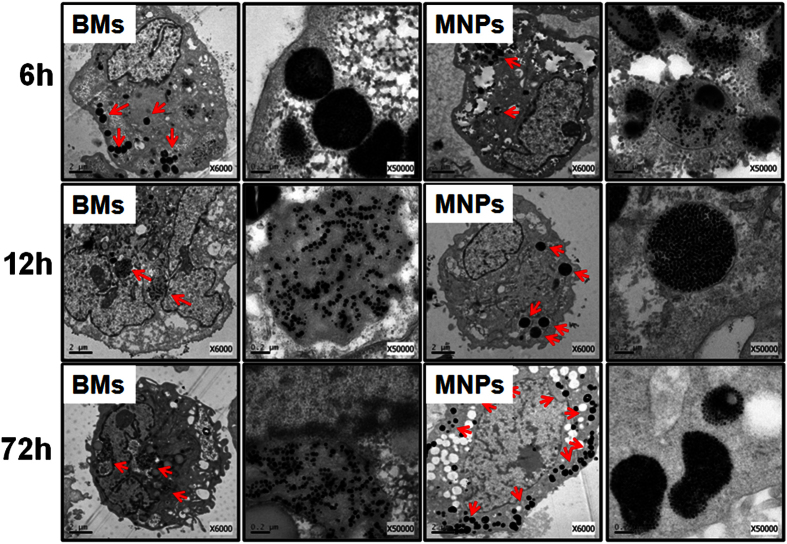
TEM images of ARPE-19 cells treated with 100 μg/mL BMs (left two columns) or MNPs (right two columns) for 6 h, 12 h and 72 h.

As shown in Fig. 2, the BMs and MNPs entered ARPE-19 cells after a 6-h exposure and agglomerated in the endocytic vesicles. The BMs displayed greater dispersion in cells compared with the MNPs, which were agglomerated by 72 h of incubation; the BM-treated cells maintained a normal morphology up to 72 h of incubation. A large number of vacuoles were observed in the MNP-treated cells after 72 h of incubation, and the number of dead cells was higher for MNP-treated cells than for BM-treated cells. When the incubation time was extended to 96 h, the BM-treated ARPE-19 cells continued to undergo cell division (Fig. S3), but only fragments of MNP-treated cells were observed. These results suggest that the MNPs were more cytotoxic than BMs against ARPE-19 cells. The BMs and MNPs were both scattered in the cytoplasm, with no organelle specificity.

#### Cell viability

**Figure 3:**
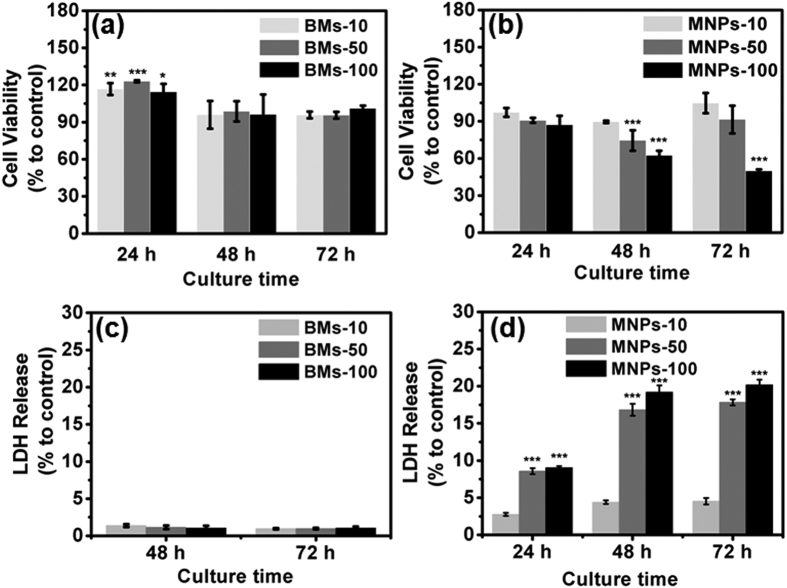
Cell survival rates of ARPE-19 cells incubated with various amounts of BMs and MNPs for 24, 48 and 72 h. The viability of the BM-treated cells (**a**) and MNP-treated cells (**b**) was determined using the CCK-8 assay. The LDH release rates of the BM-treated cells (**c**) and MNP-treated cells (**d**) were also determined.

The cell viability of ARPE-19 cells in the presence of BMs or MNPs in the culture medium was determined by detecting the activities of intracellular dehydrogenases (CCK-8 assay) and membrane integrity (LDH test). BMs strikingly promoted the growth of ARPE-19 cells after 24 h of incubation (Fig. 3a). The cell survival rate was greater than 90%, even after incubation with 100 μg/mL BMs for 72 h, with an IC50 of 146 mg/mL. In addition, the BMs also strongly promoted the viability of other ocular cells, such as the cone cell line 661W (Fig. S4). The survival rates of ARPE-19 cells significantly decreased with increasing MNP concentrations and culture times. As shown in Fig. 3b, the cell survival rate was less than 50% after incubation with 100 μg/mL MNPs for 72 h, and the IC50 was 95.7 μg/mL.

Furthermore, the results of the LDH assay indicated that the BMs did not induce of LDH in ARPE-19 cells within 24 h, based on the negative values observed in the LDH test. However, after exposure for 24, 48 or 72 h, LDH levels were significantly higher in MNP-treated cells than in control cells, even at a concentration of 10 μg/mL (Fig. 3d). By contrast, the BMs had a negligible influence on LDH leakage (Fig. 3c). These results indicate that the cytotoxicity of the BMs was much lower than that of the MNPs.

### Genotoxicity of BMs and MNPs

#### Induction of DNA damage in ARPE-19 cells by BMs or MNPs

**Figure 4:**
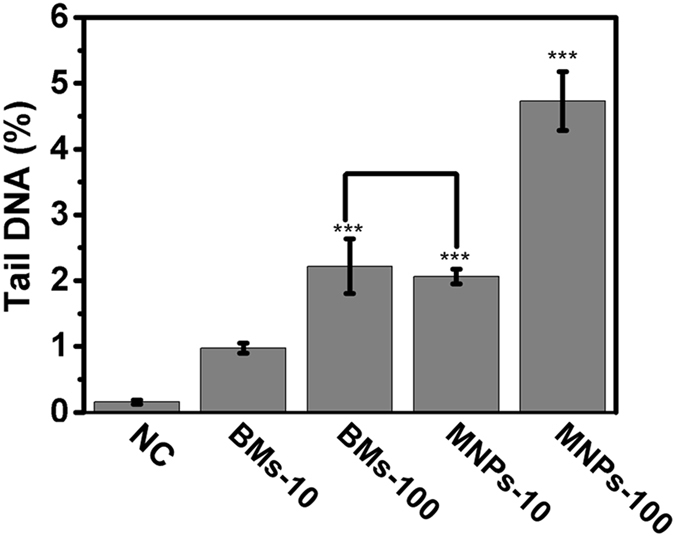
DNA damage in BM- or MNP-treated ARPE-19 cells was determined by Comet Assays. The percent DNA in the tails of the ARPE-19 cells was calculated using CASP software.

The induction of DNA damage by BMs or MNPs in ARPE-19 cells was assessed by the alkaline Comet Assay (Fig. S5). According to the DNA damage tail percentage in ARPE-19 cells (Fig. 4), both BMs and MNPs induced DNA damage in ARPE-19 cells. However, MNPs caused greater DNA damage than BMs; the tail percentage at 100 μg/mL MNPs was twofold higher than that for cells treated with BMs at the same concentration (Fig. 4).

#### ROS levels

**Figure 5:**
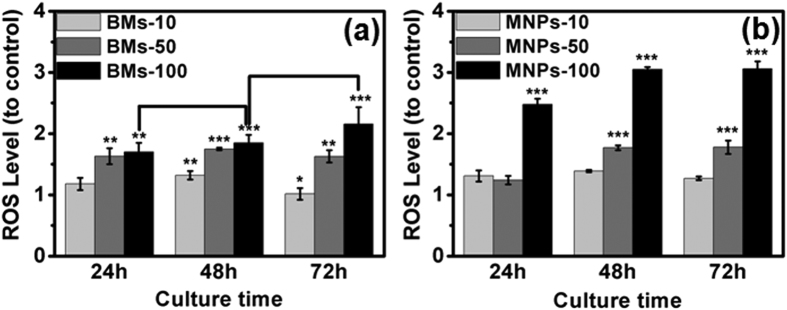
The effects of BMs (**a**) and MNPs (**b**) on ROS levels in ARPE-19 cells.

Excess reactive oxygen species (ROS) induce oxidative stress, one of the most important causes of DNA damage in cells^16^. Exposure to BMs and MNPs induced oxidative stress in ARPE-19 cells (Fig. 5), and the level of ROS production increased with increasing concentrations of BMs or MNPs. However, ROS levels did not increase with BM exposure time. For example, when treated with 100 μg/mL BMs for 24 h, the ROS level in the BM-treated cells was 1.7-fold higher than in the control. At longer incubation times of 48 and 72 h, ROS levels were 1.85- and 2-fold higher, respectively, than in the control, but these increases with time were not significant. ROS levels were 1.5-fold higher in 100 μg/mL MNP-treated cells than in BM-treated cells at 72 h (Fig. S6). In the positive control (normal cells cultured with medium containing 500 mM H_2_O_2_ for 30 min), the ROS level was 4.97-fold higher than in the control (data not shown), much higher than the ROS levels in BM- and MNP-treated cells under the same conditions.

#### Cell apoptosis

**Figure 6:**
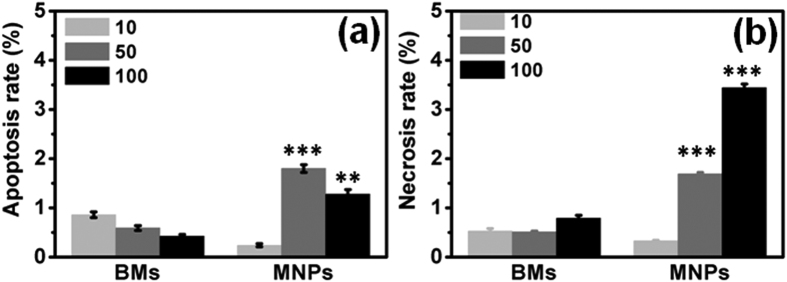
Summary of the apoptosis rates (**a**) and necrosis rates (**b**) of ARPE-19 cells after exposure to BMs and MNPs for 24 h.

The apoptotic and necrotic rates of BM- and MNP-treated ARPE-19 cells were further studied by fluorescence-activated cell sorting (FACS) after staining of dead and apoptotic cells with propidium iodide (PI) and Annexin V-FITC, respectively. As shown in Fig. S7, cells undergoing the early stage of apoptosis were observed in the lower right section (PI^−^/FITC^+^), indicating the release of the phospholipid phosphatidylserine (PS). The cells in the upper right section were PI^+^/FITC^+^ and correspond to the end stage of apoptosis, necrosis or dead cells. The rates of apoptosis of MNP-treated cells were much higher than those of BM-treated cells (Fig. 6). For example, the apoptosis rate of ARPE-19 cells treated with 100 μg/mL MNPs was 1.28%, nearly 3 times that of the control and cells treated with an identical concentration of BMs. Moreover, the MNPs also significantly increased the rate of necrosis. For example, 100 μg/mL MNPs induced necrosis in 3.44% of cells, a rate fourfold higher than that induced by BMs. Based on the results of Comet assays and ROS detection, both BMs and MNPs induce ROS-dependent DNA damage, leading to apoptosis and necrosis of ARPE-19 cells. Compared with the MNPs, the BMs exhibited much weaker genotoxicity.

**Figure 7:**
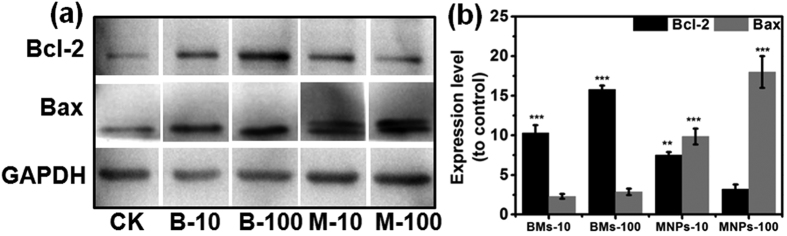
The expression of Bcl-2 and Bax in the BM- and MNP-treated cells was determined by western blotting. (**a**) Images of the western blots; (**b**) relative quantities of Bcl-2 and Bax.

The levels of apoptosis-related proteins were further determined by western blotting. Bcl-2 and Bax suppress and facilitate apoptosis, respectively. Knudson *et al.*^17^ observed that a single copy of Bax promoted apoptosis, whereas overexpression of Bcl-2 still repressed apoptosis. As shown in Fig. 7, Bcl-2 protein expression was significantly increased in BM-treated cells; this increase may suppress apoptosis in a variety of cells^18^. However, Bax protein expression was higher in MNP-treated cells than in BM-treated cells; this increase may accelerate programmed cell death by binding to and antagonizing the apoptosis repressor Bcl-2^18^. Furthermore, the ratios of Bcl-2 and Bax expression were opposing in BM- and MNP-treated cells. Both BMs and MNPs induced apoptosis in ARPE-19 cells; however, the BM-treated cells tended to suppress apoptosis progression and returned to normal conditions, whereas the MNP-treated cells entered late apoptosis, and apoptosis progression could not be suppressed. These results further indicate that the BMs were less cytotoxic than the MNPs.

## Conclusions

The cytotoxicity and genotoxicity of BMs and MNPs with similar morphologies, surface groups and size characteristics in ARPE-19 cells were investigated for the first time. Both BMs and MNPs entered ARPE-19 cells by endocytosis. BM-treated cells maintained normal cell morphology for up to 72 h, whereas most of the MNP-treated cells appeared to undergo autolysis. The BMs affected the cell survival rate and cell membrane integrity slightly, whereas a high concentration of MNPs significantly decreased the survival rate of ARPE-19 cells, as supported by both CCK-8 and LDH tests. The BMs and MNPs both featured surface amino groups. However, the BM membrane had extra carboxyl and amide groups, which enhanced the hydrophilicity and biocompatibility of the BMs. In addition, both BMs and MNPs induced DNA damage. However, DNA damage due to increased ROS production was reduced in BM-treated cells compared to MNP-treated cells. The BMs and MNPs also induced apoptosis, but the high concentration of MNPs caused obvious cell necrosis. Western blot analysis revealed that the BMs significantly induced the expression of Bcl-2 and the MNPs significantly induced the expression of Bax. Bcl-2 and Bax suppress and facilitate apoptosis, respectively. Therefore, both BMs and MNPs caused DNA damage in ARPE-19 cells, but the damage caused by the BMs was controllable and limited and could be reversed by cellular self-repair. By contrast, the DNA damage caused by the MNPs was serious and difficult to repair; thus, the cells underwent apoptosis and eliminated the damaged cells. Therefore, the MNPs displayed much stronger genotoxicity than the BMs. In summary, the BMs displayed excellent cytocompatibility and reversible genotoxicity toward ARPE-19 cells, indicating great potential for the treatment of ocular diseases.

## Methods

### Reagents and Materials

The BMs were isolated from *M. gryphiswaldense* MSR-1 according to a previously published method^19^. MSR-1 strains in logarithmic phase were collected by centrifugation. The cells were resuspended in 10 mL of PBS (0.01 M, pH 7.4), and the cell membranes were disrupted by ultrasonication (SCIENTZ, China) at 300 W. The crude BM extract was collected for 24 h using magnets. The BMs were continuously cleaned by ultrasonication (KQ500D, China) in PBS (0.01 M, pH 7.5) at 100 W for 1 h and collected with magnets for 2–3 h. The ultrasonic cleaning and magnet adsorption steps were repeated until the ultraviolet (UV) absorption of the supernatants was zero at both 260 and 280 nm. The BMs were freeze dried and stored at −20 °C. The supernatants of each step and the BM membranes were separated by SDS-PAGE and stained with Coomassie blue G250. *Han*^20^ reported that 1 mg of BMs (extracted from wild type MSR-1) contains 0.605 mg of iron, and thus the concentration of BMs was annotated by the iron content in subsequent studies. The iron oxide (II, III) magnetite nanoparticle solution (MNPs, CAS: 1317-61-9) was purchased from Sigma-Aldrich and contained nanoparticles ranging in size from 28 to 32 nm. One milliliter of MNP solution contained 1 mg of iron. For subsequent cell biocompatibility studies, the BMs and MNPs were sterilized by irradiation (15 kGy)^21^ and high-pressure steam, respectively.

The BMs and MNPs were characterized by FTIR (Thermo Nicolet 6700) and Zeta Plus (Zetasizer Nano ZS 90), and the magnetic properties were determined with a vibrating sample magnetometer Model 3900 (Princeton Measurements Corporation, USA, sensitivity 5.0 × 10^−10 ^Am^2^). The morphologies and sizes of both the BMs and MNPs were observed by HRTEM (JEOL).

### Cell Culture

ARPE-19 cells and 661W cells were cultured in DMEM/F12 (Gibco) supplemented with 10% (V/V) qualified fetal bovine serum (Gibco) and gentamicin (50 μg/mL, Gibco) at 10% CO_2_ and 37 °C.

### Transmission Electron Microscopy (TEM)

ARPE-19 cells were cultured in an incubator with 100 μg/mL BMs or MNPs for various times (2, 6, 12, 24, 48 and 72 h). The cells were fixed in 2.5% glutaraldehyde solution as described previously^22^. After embedding, the cells were sliced into ultra-thin sections and stained with toluidine blue for TEM observations.

### Cell Viability

Cell Counting Kit-8 (CCK-8, Dojindo) was used to determine cell viability. The cells were seeded in 96-well plates at a density of 3,000 cells/well and incubated for 24 h. The cells were cultured in an incubator with various concentrations (10, 50 and 100 μg/mL) of BMs or MNPs. Cells cultured in medium without BMs or MNPs were used as a negative control. After 24, 48 and 72 h of incubation, the culture medium was replaced with 100 μL of CCK-8 solution diluted in culture medium at a V/V ratio of 1:10 and incubated for an additional 3 h at 37 °C. The optical density (OD) of each well at 450 nm was recorded by a SpectraMax M5 (Molecular Devices). Cell viability was expressed as the following percentage: (OD_test_ − OD_blank_)/(OD_negative_ − OD_blank_). OD_blank_ was the OD value of culture medium without ARPE-19 cells. The IC50 value was calculated using Bliss’s method^23^.

### Membrane Integrity

An LDH test kit (CytoTox 96^®^ Non-Radioactive Cytotoxicity Assay, Promega Co.) was used to evaluate cell membrane integrity. ARPE-19 cells were seeded in 96-well plates (5,000 cells per well) and incubated for 24 h. Then, the cells were treated with various concentrations (10, 50, and 100 μg/mL) of BMs or MNPs. Cells cultured in medium without BMs or MNPs were used as a negative control. After 24, 48 and 72 h of incubation, the 96-well plates were centrifuged at 250 *g* for 4 min. The LDH maximum leakage control (positive control) was prepared by adding 10 μL of lysis solution to the control cells 45 min prior to centrifugation. After centrifugation, 50 μL of supernatant from each well was transferred to a new 96-well plate for the LDH assay according to the instructions provided with the kit. The absorbance at 490 nm was recorded by a SpectraMax M5. LDH leakage was expressed as the following percentage: (OD_test_ − OD_blank_)/(OD_positive_ − OD_blank_). OD_blank_ was the OD of the culture medium without ARPE-19 cells.

### Reactive Oxygen Species (ROS) Assay

ARPE-19 cells were plated in black 96-well plates (Clear bottom, Costar 3603) at 10,000 cells per well and incubated for 24 h. The cells were cultured in an incubator with various concentrations (10, 50, and 100 μg/mL) of BMs or MNPs for 24, 48 and 72 h. The oxidant-sensitive dye DCFH-DA was used to detect ROS (Reactive Oxygen Species Assay Kit, Beyotime Institute of Biotechnology, China). For all cells, the culture medium was then replaced with 100 μL of new culture medium (without fetal bovine serum) containing 10 μM of DCFH-DA and incubated for 20 min at 37 °C in the dark. The cells were washed with DMEM/F12 (without fetal bovine serum) three times. Positive controls were prepared by culturing the normal cells with culture medium containing 500 mM H_2_O_2_ for 30 min after the cells were labeled with DCFH-DA, and the negative controls were incubated in normal culture medium. The fluorescence intensity was detected by SpectraMax M5 at an excitation wavelength of 488 nm and an emission wavelength of 525 nm in bottom-read mode. The ROS level was expressed as the following ratio: (OD_test_ − OD_blank_)/(OD_negative_ − OD_blank_). OD_blank_ was the fluorescence intensity of the culture medium without ARPE-19 cells.

### Apoptosis Assay

Apoptotic and necrotic cells were detected using an Apoptosis kit (FITC Annexin V Apoptosis Detection Kit I, BD Biosciences, USA). ARPE-19 cells were plated in 25-cm^2^ cell-culture flasks at 1 × 10^6^ cells per flask and incubated for 24 h. Various concentrations of BMs and MNPs (10, 50 and 100 μg/mL) were added to the cells and incubated for an additional 24 h. The negative control was prepared in medium with no additives. The cells were stained with Annexin V-FITC and PI according to the manual provided with the kit. The stained cells were analyzed by FACS (FACSCalibur, BD Biosciences, USA).

### Alkaline Comet Assay

The alkaline Comet assay reagent kit (TREVIGEN, Catalog # 4250-050-K) was used to evaluate DNA damage in cells, including single- and double-strand breaks. ARPE-19 cells were cultured in polystyrene 6-well tissue culture plates (Corning) at a density of 1 × 10^5^ cells per well for 24 h. The cells were continuously cultured in the incubator with various concentrations of BMs or MNPs (10 or 200 μg/mL) for 24 h. Cells cultured in medium with or without 500 mM H_2_O_2_ for 30 min were used as the positive or negative controls, respectively. The alkaline Comet assay was performed according to the instructions in the manual. DNA was visualized by staining the slides with the fluorescent DNA binding dye SYBR Green I and observed with a fluorescence microscope (OLYMPUS, IX81). The percent DNA in the tail was scored using the software of the Comet Assay Software Project (CASP). Two hundred cells were analyzed per sample (two duplicate sample slides, 100 randomly selected cells scored per slide).

### Western Blot

ARPE-19 cells were grown on polystyrene 6-well tissue culture plates at a density of 2.5 × 10^5^ cells/well and incubated for 24 h. The cells were continuously cultured in the incubator with various concentrations of BMs or MNPs (10 or 100 μg/mL) for 24 h. Cells cultured in medium without additives were used as the negative control. The ARPE-19 cells were harvested and lysed in western cracking buffer (Beyotime) for 5 min. The total protein solutions were collected by centrifugation at 14,000 × *g* for 15 min and concentrated with an Amicon ultra centrifugal filter (Millipore, 0.5 mL, 10 kDa) at 4 °C. The concentrations of the proteins were determined by the BCA Protein Assay Kit (Thermo) according to the instructions provided by the supplier.

Western blots were performed to analyze Bcl-2 or Bax expression levels by probing the membranes with Bcl-2 (Abcam, Cat# ab32124) or Bax (Abcam, Cat# ab32503) rabbit polyclonal antibodies, respectively. The samples were blotted with Goat Anti-Rabbit IgG H&L (HRP) secondary antibodies (Abcam, Cat# ab97051) after the primary antibody incubation. GAPDH was used as the loading control by probing the membrane with the HRP-anti-GAPDH monoclonal antibody (KANGCHEN, Cat# KC-5G5). A chemiluminescent substrate kit (SuperSignal West Dura Extended Duration Substrate, Thermo) was used to detect HRP on the immunoblots. The relative peaks of the bands (normalized to the band of GAPDH) were calculated using Image Lab software (Bio-Rad Laboratories, Inc.).

### Statistical analysis

Six parallel tests were conducted for each condition. All data are presented as the means and standard deviations (means ± SD). Because all data were normally distributed (the skewness and kurtosis values of all data were calculated by SPSS), the significance (p value) was calculated using unpaired, two-tailed Student’s *t*-tests with unequal variance. The symbol “*” denotes statistical significance (* ≤ 0.05, ** ≤ 0.01, *** ≤ 0.001) compared with the negative control, and p < 0.05 was considered significant.

## Additional Information

**How to cite this article**: Qi, L. *et al.* Cytotoxicity and genotoxicity of bacterial magnetosomes against human retinal pigment epithelium cells. *Sci. Rep.* **6**, 26961; doi: 10.1038/srep26961 (2016).

## Supplementary Material

Supplementary Information
