# Supplementary material for: Cytotoxicity and genotoxicity of bacterial magnetosomes against human retinal pigment epithelium cells
Source: Sci Rep. 2016 Jun 1;6:26961. doi: 10.1038/srep26961 (PMC4887902; doi:10.1038/srep26961)
Supplement: Supplementary Information [file srep26961-s1.pdf]

Supplementary Information

Cytotoxicity and genotoxicity of bacterial magnetosomes against human retinal pigment epithelium cells

Lei Qi[a], Xiujuan Lv[a], Tongwei Zhang[c, d], Peina Jia[a], Ruiying Yan[a], Shuli Li[e], Ruitao Zou[a], Yuhua Xue[a, *], Liming Dai[a, b,]**

[a]. Institute of Advanced Materials for Nano-Bio Applications, School of Ophthalmology & Optometry, Eye Hospital, Wenzhou Medical University, 268 Xue yuan Xi Road, Wenzhou, Zhejiang 325027, China

[b]. Center of Advanced Science and Engineering for Carbon (Case4Carbon), Department of Macromolecular Science and Engineering, Case Western Reserve University, Cleveland, OH 44106,United States

[c]. Biogeomagnetism Group, Paleomagnetism and Geochronology Laboratory, Key Laboratory of Earth and Planetary Physics, Institute of Geology and Geophysics, Chinese Academy of Sciences, Beijing 100029, China

[d]. France-China Bio-Mineralization and Nanostructures Laboratory, Chinese Academy of Sciences, Beijing 100029, China

[e]. Department of Microbiology, College of Biological Sciences, China Agricultural University,Beijing,100193, China

* Corresponding author.

**Corresponding author.

E-mail addresses: yuhua_xue@hotmail.com (Y. Xue), liming.dai@hotmail.com (L. Dai)

Results

(a)	A260	A280
1	90.9	61.58
2	7.98	5.76
3	0.81	0.61
4	0.207	0.142
5	0	0

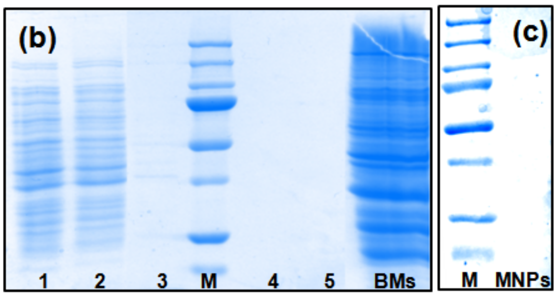

Figure S1. Isolation of BMs from MSR-1. (a) The UV absorptions of the extractive supernatants at 260 and 280 nm from step 1 to 5. (b) Determine the proteins of the extractive supernatants from step 1 to 5 and the surface of BMs. 1 and 2 were diluted 100 and 10 folds by PBS (0.01M, pH 7.4) respectively. (c) Detection the surface of MNPs by SDS-PAGE. The "M" of image (b) and (c) is the protein marker corresponding to 250, 150, 100, 75, 50, 37, 25, 20 kDa from up to down.

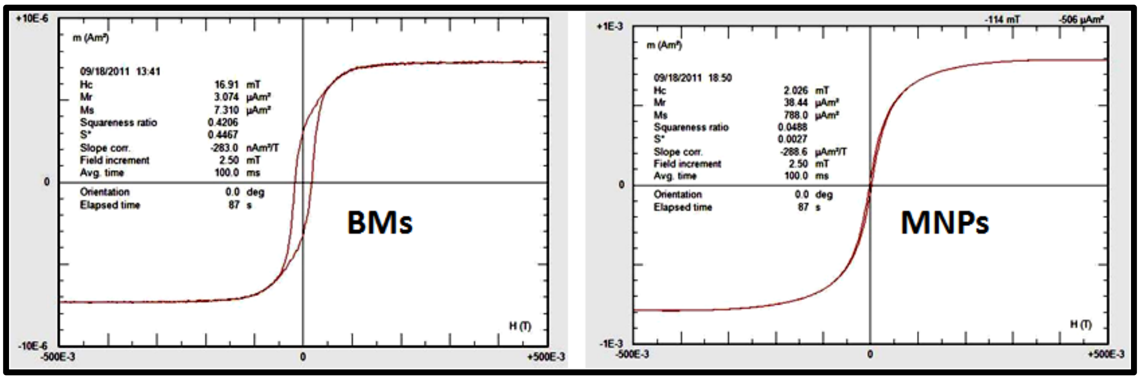

Figure S2. Normalized hysteresis loops for BMs and MNPs.

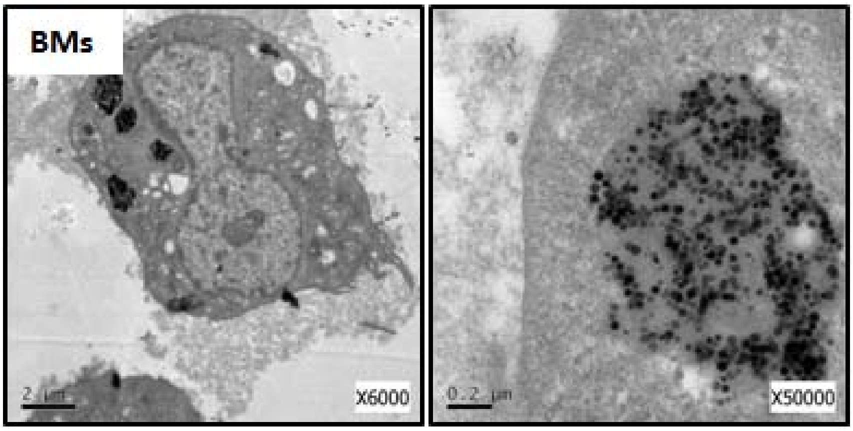

Figure S3. TEM images of ARPE-19 cells induced by BMs in the concentration of 100 μg/mL for 96 h.

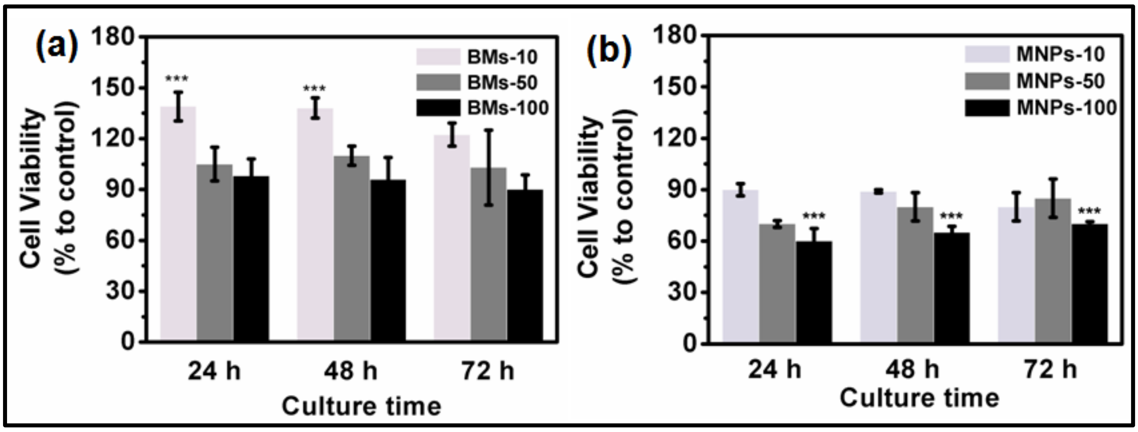

Figure S4. Cell viability of BMs-treated 661W cells (a) and MNPs-treated 661W cells (b) were determined by CCK-8 detection.

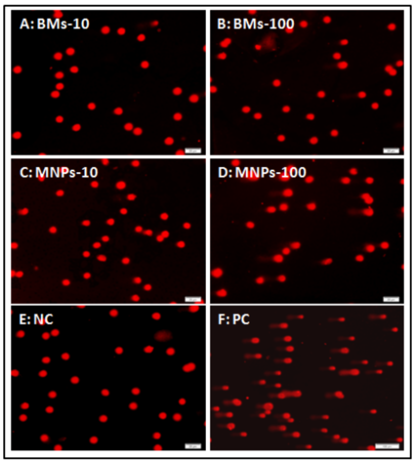

Figure S5. Example images of comet tails for ARPE-19 cells: cells treated with 10 μg/mL BMs (A), 100 μg/mL BMs (B), 10 μg/mL MNPs (C) 100 μg/mL MNPs (D), negative control (E), positive control (F).

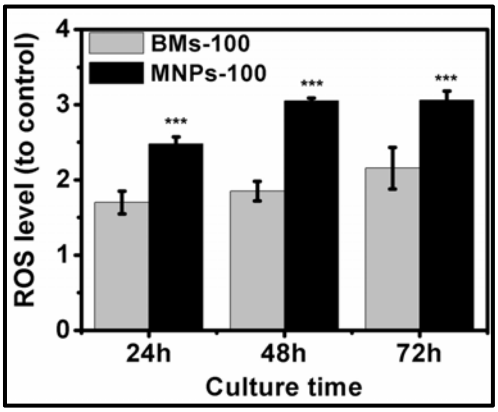

Figure S6. Comparison of the ROS level in the ARPE-19 cells induced by BMs and MNPs (100 μg/mL).

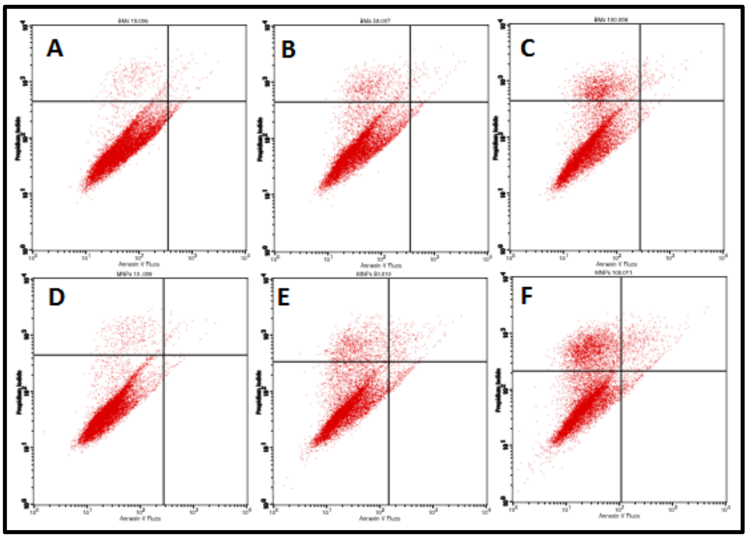

Figure S7. FACS diagrams of ARPE-19 cells exposed to BMs for 24h with the concentration of 10 μg/mL (A), 50 μg/mL (B), 100 μg/mL (C), to MNPs for 24 h with the concentration of 10 μg/mL (D), 50 μg/mL (E), 100 μg/mL (F); Y-axis and X axis indicated the PI and Annexin V FITC stained cells, respectively, in images of A to F
